# Risk of peripheral facial palsy following parenteral inactivated influenza vaccination in the elderly Chinese population

**DOI:** 10.3389/fpubh.2023.1047391

**Published:** 2023-01-24

**Authors:** Tianchi Yang, Rui Ma, Lixia Ye, Qiuhong Mei, Jianmei Wang, Yueyi Feng, Shaoying Zhou, Xingqiang Pan, Danbiao Hu, Dandan Zhang

**Affiliations:** ^1^Immunization Center, Ningbo Municipal Center for Disease Control and Prevention, Ningbo, China; ^2^Immunization Center, Ninghai County Center for Disease Control and Prevention, Ningbo, China

**Keywords:** peripheral facial palsy, inactivated influenza vaccination, self-controlled case series study, elderly, relative incidence ratio

## Abstract

**Background:**

Concern about the risk of peripheral facial palsy (PFP) following vaccination is one reason for hesitancy in influenza vaccination. However, the association between the flu vaccine and PFP is still controversial, and further evidence is urgently needed.

**Methods:**

This self-controlled case series study evaluated PFP risk following inactivated influenza vaccine in the elderly using a large linked database in Ningbo, China. Relative incidence ratios (RIRs) and 95% confidence intervals (CIs) estimated using conditional Poisson regression were utilized to determine whether the risk of PFP was increased after vaccination.

**Results:**

This study included 467 episodes, which occurred in 244 females and 220 males. One hundred twenty-four episodes happened within 1–91 days after vaccination, accounting for 26.7%. The adjusted RIRs within 1–30 days, 31–60 days, 61–91 days, and 1–91 days after influenza vaccination were 0.95 (95% CI 0.69–1.30), 1.08 (95% CI 0.78–1.49), 1.01 (95% CI 0.70–1.45), and 1.00 (95% CI 0.81–1.24), respectively. Similar results were found in subgroup analyses and sensitivity analyses.

**Conclusions:**

Influenza vaccination does not increase PFP risk in the elderly population. This finding provides evidence to overcome concerns about facial paralysis after influenza vaccination.

## 1. Introduction

Peripheral facial palsy (PFP) is the partial or total loss of function of some or all of the tissues innervated by the facial nerve, of which Bell's palsy is a type of acute peripheral facial paralysis. The pathogenesis of the disease is still unclear, and it may be related to viral infection, autoimmune diseases, and neurological ischemia (1).

In 2004, a Swiss study reported an increased risk of Bell's palsy with inactivated intranasal influenza vaccine, directly triggering public concerns about the safety of influenza vaccines at the time (2). Since then, studies have suggested a possible link between influenza vaccination and facial paralysis (3–5). However, some studies have given conflicting results that influenza vaccines do not increase the risk of PFP (6–9). In the past 2 years, the case reports of facial paralysis after the 2019-nCoV vaccination have aroused the issue of vaccination and facial paralysis again (10–14). To our knowledge, so far, the association between influenza vaccination and PFP remains inconclusive, and further evidence is urgently needed (15). In addition, little is known about the risk of PFP following influenza vaccination in elderly Chinese.

According to the World Health Organization, the elderly are the highest-risk priority group for seasonal influenza vaccination. However, worries about vaccine-induced immune-mediated adverse reactions directly influence vaccination willingness (16). In reality, the coverage rate of influenza vaccine among the elderly population in most countries in the world is not high, far below the 75% vaccination coverage recommended by the World Health Organization (17).

During the 2020/2021 flu season, Ningbo, China implemented a free influenza vaccination campaign for the elderly. From September 2020 to February 2021, 278,959 people over the age of 70 were vaccinated against influenza, with a coverage rate of nearly 46%. In the current study, we used a self-controlled case series (SCCS) design for the first time to assess the risk of PFP after influenza vaccination in the elderly Chinese population through an electronic database linking vaccination records with regional diagnosis and treatment information.

## 2. Materials and methods

### 2.1. Data sources

Ningbo is a port city on the southeast coast of China and is geographically adjacent to Shanghai. In 2020, the population was over 9.4 million. The Ningbo Regional Health Care Database (NRHCD), a standardized medical information network in Ningbo, China, collects and integrates electronic health records from hospitals and community clinics in the jurisdiction. In 2016, the platform reached the top level in the regional health information interconnection standardization and maturity measurement of the National Health Commission (18–20). By 2019, the platform covered all 65 public hospitals and 154 primary medical institutions. Vaccination data from all clinics in the region are recorded electronically and transmitted in real time to the Ningbo Vaccination Registration Database (NVRD).

In this study, the NRHCD provided information on outpatient cases of PFP diagnosed between June 2020 and April 2021. The NVRD provided influenza vaccination information for the 2020/2021 influenza season (the last vaccination date was February 27, 2021). Both medical visits and vaccinations in the study area require registration of citizens' unique personal identification numbers, so two databases can be linked at an individual level through identification numbers. The linked records were deidentified before they were further processed and analyzed. This study was approved by the Ethical Review Committee of the Center for Disease Control and Prevention of Ningbo (IRB. No: Y2021005). The requirement for informed consent was waived.

### 2.2. Study population

This study was restricted to outpatients aged 70 years and older who had at least one episode of PFP between June 1, 2020, and April 30, 2021. Analysis was further limited to patients who received parenteral trivalent inactivated influenza vaccine (IIV3, containing subtypes A/H1N1, A/H3N2, and B/Victoria) during the 2020/2021 influenza season. The date of onset of the first neurological symptom reported in the outpatient medical record was considered the case index date. If individuals had multiple episodes during the observation period, any recurrence within 6 months of the previous seizure was considered part of the same event. The study exclusion criteria were as follows: floating population, registered with NRHCD after June 1, 2020, missing vaccination date or vaccinated with other vaccines than influenza vaccine during the observation period.

PFP cases were identified by using the International Classification of Diseases version 10 (ICD-10) code G51.0 (Bell's palsy) and G51.9 (Disorder of facial nerve, unspecified). For these cases, we retrospectively reviewed the medical records 7 days before the onset of PFP and confirmed that there was no herpes virus infection, influenza, other acute infectious diseases, cerebral hemorrhage, etc.

### 2.3. Statistical analysis

We compared the risk of PFP after IIV3 vaccination in a risk period with the risk in a control period using a SCCS method. Referring to previous studies (7), the risk period in the primary analysis was defined as 1–91 days following vaccination, and the remaining observation period was defined as a control period. To avoid the impact of pre-vaccination health effects and day-of-administration chance records on background risk estimates (7, 21), we considered the 14 days before vaccination and the day of vaccination as separate risk periods. The seasonal effect was adjusted for winter-spring (from December to April) and summer-autumn (from June to November). Subgroup analyses were performed by age group and PFP type. Moreover, two sensitivity analyses were conducted: (1) using risk intervals of 1–14 days, 1–77 days, and 1–105 days following vaccination. (2) Only selecting the post-vaccination control period.

Relative incidence ratios (RIRs) and 95% confidence intervals (CIs) were estimated using conditional Poisson regression. A two-sided *p* < 0.05 indicated statistical significance. All analyses were performed by using R software, version 4.2.0.

### 2.4. Sample size

Under the assumption of 11 months of observation duration, 45.6% vaccine coverage, 80% power, and a type 1 alpha level of 0.05, 450 cases were required to identify a RIR of 1.5 or greater during 1–91 days following the IIV3 immunization.

## 3. Results

### 3.1. Characteristics of the elderly with PFP

**Figure 1:**
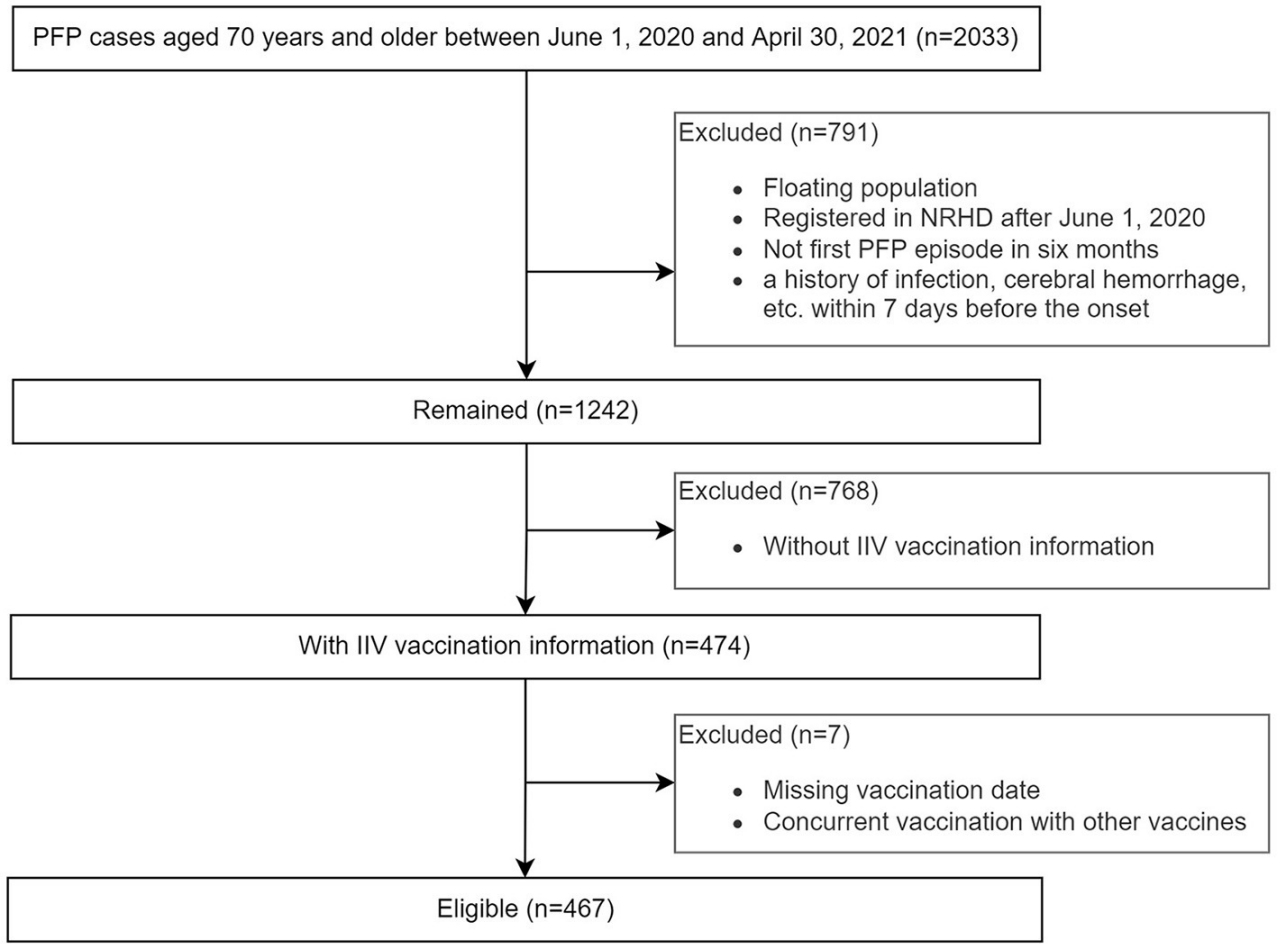
Flowchart of the study participants.

This study included 467 episodes, which occurred in 244 females and 220 males (Figure 1; Table 1). One hundred twenty-four episodes occurred within 1–91 days after IIV3 vaccination, accounting for 26.7%. Three individuals had two episodes. The age of onset ranged from 70 to 95 years, with a median age of 74 years. There were no significant differences in the age of onset and the proportion of PFP episodes within 1–91 days after IIV3 vaccination between males and females.

**Table 1:** Characteristics of the elderly with PFP in the study.

**Characteristic**	**No**.	**Median onset age, years (IQR)**	**During 1–91 days after IIV3 vaccination, no. (%)**
Male	220	75 (72, 79)	59 (26.8)
Female	244	74 (72, 77)	65 (26.6)
Total	464	74 (72, 78)	124 (26.7)

PFP, peripheral facial palsy; IQR, interquartile range; IIV3, trivalent inactivated influenza vaccine.

### 3.2. Risk of PFP following IIV3 vaccination

The risk of PFP within 1–91 days after influenza vaccination was not higher than the background risk. We divided the risk period into three 30-day periods and estimated the relative incidence ratio for each period. The results showed no significant risk increase was found in any period (Table 2). In addition, the risk on the day of vaccination and 14 days before vaccination was also comparable to the risk in the background period. Although PFP episodes did not show significant seasonality, we adjusted relative incidence ratio by season in our analysis because influenza vaccination was highly seasonal. Table 2 suggests that there is some confounding by season, but this does not change the interpretation of the results.

**Table 2:** The relative incidence ratio of PFP after IIV3 vaccination.

**Risk period**	**No. of episodes**	**RIR (95% CI)**	Adjusted RIR (95% CI)^*^
−14 to −1 days	15	0.75 (0.45–1.25)	0.69 (0.41–1.16)
Day 0	1	0.70 (0.10–4.97)	0.65 (0.09–4.64)
1 to 91 days	124	0.95 (0.77–1.17)	1.00 (0.81–1.24)
1 to 30 days	43	1.00 (0.73–1.37)	0.95 (0.69–1.30)
31 to 60 days	43	1.00 (0.73–1.37)	1.08 (0.78–1.49)
61 to 91 days	38	0.85 (0.61–1.20)	1.01 (0.70–1.45)

PFP, peripheral facial palsy; RIR, relative incidence ratio; CI, confidence interval.

* Adjusted for seasonality.

Subgroup analysis stratified by age group (Supplementary Table 1) and PFP type (Supplementary Table 2) also showed that the relative incidence ratio of PFP did not increase in each risk period after vaccination.

### 3.3. Sensitivity analysis

The sensitivity analysis results were consistent with those of the primary analysis regardless of whether a narrower or wider risk period was used, or only the post-vaccination control period was selected (Table 3).

**Table 3:** Sensitivity analysis for relative incidence ratio of PFP after IIV3 vaccination.

**Risk period**	**No. of episodes**	**Sensitivity analysis one^a^** Adjusted RIR (95% CI)^*^	**Sensitivity analysis two^b^** Adjusted RIR (95% CI)^*^
1–14 days	25	1.19 (0.79–1.78)	1.20 (0.74–1.94)
1–77 days	101	0.93 (0.75–1.17)	0.80 (0.57–1.13)
1–105 days	136	0.95 (0.77–1.17)	0.85 (0.64–1.14)

PFP, peripheral facial palsy; RIR, relative incidence ratio; CI, confidence interval.

a Using narrower or wider post-vaccination intervals;

b only selecting the post-vaccination control period.

* Adjusted for seasonality.

## 4. Discussion

During the 2020/2021 influenza season, no increased risk of PFP was observed within 91 days after IIV3 vaccination among Chinese adults aged 70 years and older. Furthermore, subgroup analyses and sensitivity analyses did not reveal an association between influenza vaccine exposure and PFP. To our knowledge, this is the first study of the relationship between influenza vaccination and PFP in an elderly Chinese population using a large linked database. Although controversial in the scientific literature (22), our study provides evidence from China to support that influenza vaccination does not significantly increase the risk of PFP.

The possibility of vaccine-induced immune-mediated adverse effects has raised concerns about a link between vaccines and peripheral facial paralysis (15). However, our data did not identify a significant association between vaccination and PFP, which is consistent with the findings of other studies also designed for SCCS (6–9). Conversely, some studies observed an increased risk of PFP following influenza vaccination (3–5, 23–25). The most influential of these was the intranasal influenza vaccine used in Switzerland in 2000–2001, which was found to significantly increase the risk of PFP in this case-control study (adjusted OR, 84.0; 95% CI 20.1–351.9) (2). However, subsequent experimental studies demonstrated that an E. coli heat-labile toxin as an adjuvant for the intranasal route of administration was responsible for PFP (23). Two disproportionate analyses and one capture-recapture analysis using the Vaccine Adverse Event Reporting System (VAERS) database showed that Bell's palsy reported following seasonal influenza vaccination or influenza A (H1N1) vaccination exceeded the criteria for a potentially relevant signal (3, 4, 24). However, due to the inherent limitations of passive surveillance systems (e.g., underreporting, lack of adequate data quality, etc.) and the exploratory nature of both analyses, the likelihood of risk needs to be further assessed through controlled studies. In addition, a population-based retrospective cohort study in Sweden suggested a slightly increased risk of PFP following H1N1 influenza vaccination (25). However, the excess risk was only found in high-risk groups for influenza (25), and potential causes could be comorbidities, viral infections, and pregnancy (25, 26).

Previous studies have suggested that pre-vaccination healthy effects and opportunistic records on the day of vaccination may lead to bias in baseline risk estimates (7, 21, 27). In this regard, we analyzed the 14 days before vaccination and the day of vaccination as separate risk periods. Unexpectedly, we did not observe a significant reduction in PFP risk before vaccination and an increase in cases due to opportunistic recording on the day of vaccination. It is not inconceivable in clinical practice. After all, a history of PFP does not constitute a contraindication to influenza vaccination.

Our study had several strengths. First, this study was conducted based on comprehensive regional health care data, which allowed us to identify 2,033 cases of PFP, although the disease is uncommon. With 467 cases of vaccinated PFP, our investigation had the power to detect an RIR of 1.5, with an α risk of 5%, >80%. Despite controversial findings from previous studies, our study provides a strong argument to support that seasonal influenza vaccination is not associated with a significant increase in PFP risk. Second, the SCCS design can be used to avoid exposure misclassification bias by including only vaccinated individuals (27). In addition, time-invariant confounders could be also adjusted because vaccines placed themselves in the control group (21, 27). Finally, we conducted several sensitivity analyses to ensure the robustness of our primary results.

However, there were still some limitations. First, we only considered outpatient PFP cases, because NRHIP did not provide information on no-visits and inpatient cases. Due to the impact on appearance and life (28, 29), even mild cases usually need to seek medical advice in time. However, hospitalization is generally not required. Moreover, according to the routine consultation process, the patient will have an outpatient consultation record before being hospitalized. Second, without clinical examination records, we could not verify whether the cases had undergone nerve conduction tests. Third, if the influenza vaccine is given concurrently with other vaccines such as pneumonia or shingles during the observation period, the safety profile may vary. Although the proportion of concurrent vaccinations is <1%, our findings do not apply to this situation. Finally, the findings may not be suitable for extrapolation to all older populations, as only those 70 years of age and older were included in the study.

In conclusion, our findings are reassuring, and we did not find evidence of an increased risk of PFP within 91 days following IIV3 vaccination. While the antigenic composition of influenza vaccines is adjusted annually to include recently circulating virus strains, it is necessary to continuously monitor the safety of vaccination in future flu seasons, especially when new manufacturing processes or adjuvants are introduced.

## Data availability statement

The raw data supporting the conclusions of this article will be made available by the authors, without undue reservation.

## Ethics statement

The studies involving human participants were reviewed and approved by Ningbo Municipal Center for Disease Control and Prevention. Written informed consent for participation was not required for this study in accordance with the national legislation and the institutional requirements.

## Author contributions

Conceptualization: TY, LY, DH, and DZ. Methodology: TY, LY, and YF. Resources: RM, SZ, and XP. Data curation: QM and JW. Writing—original draft preparation: TY. Project administration: TY, DH, and DZ. Writing—review and editing: All authors. All authors have read and agreed to the manuscript.
