# Supplementary material for: Risk of peripheral facial palsy following parenteral inactivated influenza vaccination in the elderly Chinese population
Source: Front Public Health. 2023 Jan 24;11:1047391. doi: 10.3389/fpubh.2023.1047391 (PMC9902766; doi:10.3389/fpubh.2023.1047391)
Supplement: Supplementary file 1 [file Table_1.DOCX]

**Title:**

Risk of peripheral facial palsy following parenteral inactivated influenza vaccination in the elderly Chinese population

**List of Supplemental Tables**

**Supplemental Table 1:** Age-specific Relative incidence ratio of PFP after IIV3 vaccination

**Supplemental Table 2:** The relative incidence ratio of two types of PFP after IIV3 vaccination

**Supplemental Table 1:** Age-specific Relative incidence ratio of PFP after IIV3 vaccination

| **Risk Period** | **Age 70-79** | |  | **Age 80+** | |
| --- | --- | --- | --- | --- | --- |
|  | **No. of episodes** | **Adjusted RIR (95%CI)*** |  | **No. of episodes** | **Adjusted RIR (95%CI)*** |
| -14 to -1 | 12 | 0.69 (0.39-1.24) |  | 3 | 0.63 (0.20-2.03) |
| Day 0 | 1 | 0.82 (0.12-5.86) |  | 0 | - |
| 1 to 91 days | 103 | 1.05 (0.83-1.33) |  | 21 | 0.82 (0.50-1.34) |
| 1 to 30 days | 32 | 0.89 (0.62-1.29) |  | 11 | 1.14 (0.59-2.19) |
| 31 to 60 days | 38 | 1.21 (0.85-1.71) |  | 5 | 0.59 (0.24-1.47) |
| 61 to 91 days | 33 | 1.11 (0.75-1.64) |  | 5 | 0.62 (0.24-1.62) |

Abbreviations: PFP, peripheral facial palsy; RIR, relative incidence ratio; CI, confidence interval.

^*^ adjusted for seasonality

**Supplemental Table 2:** The relative incidence ratio of two types of PFP after IIV3 vaccination.

| **Risk Period** | **Bell’s palsy** | |  | **Disorder of facial nerve, unspecified** | |
| --- | --- | --- | --- | --- | --- |
|  | **No. of episodes** | **Adjusted RIR (95%CI)*** |  | **No. of episodes** | **Adjusted RIR (95%CI)*** |
| -14 to -1 | 6 | 0.66 (0.29-1.52) |  | 9 | 0.68 (0.35-1.32) |
| Day 0 | 0 | - |  | 1 | 1.07 (0.15-7.63) |
| 1 to 91 days | 53 | 0.98 (0.71-1.36) |  | 71 | 1.02 (0.78-1.35) |
| 1 to 30 days | 18 | 1.04 (0.63-1.71) |  | 24 | 0.91 (0.59-1.39) |
| 31 to 60 days | 18 | 1.01 (0.61-1.66) |  | 25 | 1.12 (0.73-1.71) |
| 61 to 91 days | 17 | 0.89 (0.52-1.52) |  | 22 | 1.10 (0.68-1.78) |

Abbreviations: PFP, peripheral facial palsy; RIR, relative incidence ratio; CI, confidence interval.

^*^ adjusted for seasonality
